# Study Protocol of SURVIVE HERoes (NCT06643585): Trastuzumab Deruxtecan for molecular relapse in HER2+/ Low early breast cancer with ctDNA positivity after primary therapy

**DOI:** 10.1371/journal.pone.0322156

**Published:** 2025-11-19

**Authors:** Kerstin Pfister, Thomas W. P. Friedl, Andreas Hartkopf, Franziska Mergel, Sophia Huesmann, Forca Mehmeti, Henning Schäffler, Angelina Fink, Tatjana Braun, Sabine Heublein, Lisa Wiesmüller, Klaus Pantel, Brigitte Rack, Peter A. Fasching, Wolfgang Janni

**Affiliations:** 1 Department of Gynecology and Obstetrics, University Hospital Ulm, Ulm, Germany; 2 Institute of Women’s Health, University Hospital Tübingen, Tübingen, Germany; 3 Department of Gynecology and Obstetrics, SLK-Kliniken Heilbronn, Heilbronn, Germany; 4 Institute of Tumor Biology, University Hospital Hamburg-Eppendorf, Hamburg, Germany; 5 Department of Gynecology and Obstetrics, University Hospital Erlangen, Erlangen, Germany; The University of Texas MD Anderson Cancer Center, UNITED STATES OF AMERICA

## Abstract

**Background:**

Current evidence on circulating tumor DNA (ctDNA) in the adjuvant setting of early breast cancer (eBC) confirms its high prognostic value. CtDNA-positive patients without radiographic signs of relapse show reduced disease-free and overall survival. Secondary adjuvant treatment intervention studies represent a new appealing therapeutic option.

**Methods:**

We present SURVIVE **HERoes**, a phase III randomized clinical trial of the potent antibody-drug conjugate trastuzumab deruxtecan versus standard of care (SoC) in patients with HER2 positive or HER2 low eBC and molecular residual or recurrent disease (ctDNA positive, cM0) after primary therapy. The primary endpoint is the ctDNA clearance rate after 12 months of therapeutic intervention. A total of 180 study participants will be enrolled and randomized in a 2:1 ratio to receive trastuzumab deruxtecan or SoC therapy. The trial is accompanied by an extensive translational research project.

**Discussion:**

Treating ctDNA positive patients without radiographic signs of recurrence is a novel approach. If SURVIVE **HERoes** and similar studies targeting MRD will be positive, this may lead the way to a new molecular understanding of breast cancer stages and individualized therapy and may open a new therapeutic window for cure.

## Introduction

Despite significant progress in breast cancer diagnostics and therapy, breast cancer still accounts for over 40,000 deaths annually in the USA alone [1]. Mortality rates are notably lower when the disease is detected at a localized stage (confined to the breast and regional lymph nodes), prior to spreading to distant organs. Five-year survival rates demonstrate this stark contrast: 99% for localized disease, 86% for regional spread, and only 27% for distant metastases [2]. These figures underscore the critical importance of early detection and treatment of low tumor burden in improving patient outcomes.

Current aftercare following diagnosis and primary treatment of early breast cancer (eBC) comprises regular examinations of breast and locoregional lymph nodes, in asymptomatic patients without screening for distant metastases through, i.e., computertomography (CT) scans [3–6]. This is based on two trials of the last century, showing no evidence of improved overall survival (OS) in the arms of intensive surveillance [7,8]. However, current aftercare neglects recent advances in the field of liquid biopsies, especially promising results in the field of circulating tumor DNA (ctDNA). ctDNA are fragments of typically 180–200 base pairs, that are released to the circulation mostly via apoptosis [9], and constitute only a small fraction of total cell free DNA (<1%, [10]). There are two fundamentally different techniques of ctDNA detection:

i. Tumor-informed: Tumor tissue from surgery or biopsy undergoes whole genome or whole exome sequencing and an individual primer panel is generated for each patient.
ii. Tumor-agnostic: ctDNA levels and methylation patterns are measured by an entity-specific primer panel without the need for sequencing of primary tumor tissue.

Recent evidence has supported the role of ctDNA as a prognostic biomarker in eBC. Several studies have shown in a retro- and prospective manner that ctDNA positivity during follow-up care is associated with limited disease-free survival (DFS) and OS [11–14]. A recent meta-analysis by Nader-Marta et al. including studies using tumor-informed or tumor-agnostic approaches has shown adjusted hazard ratios (ctDNA positive vs ctDNA negative during follow-up) of 7.20 (95% CI 2.85–18.18) for DFS and 5.64 (95% CI 1.46–21.82) for OS [15].

Furthermore, recent data from a retrospective analysis of ctDNA samples from the MonarchE study underline the potential of repeated ctDNA measurements as a therapy-guiding tool. The MonarchE study evaluated the efficacy of Abemaciclib with endocrine therapy in the adjuvant setting of patients with high-risk hormone receptor positive breast cancer [16]. Loi et al. could show that patients with initial ctDNA positivity that became ctDNA negative during treatment had a significantly lower invasive-disease free survival (IDFS) rate compared to patients with persisting ctDNA positivity (10 of 24 patients, 42% vs 34 of 34 patients, 100%) [17]. Despite the limited number of participants and the retrospective approach of the analysis, this important result confirms the great potential of ctDNA as a highly prognostic biomarker in breast cancer aftercare.

Trastuzumab deruxtecan (T-DXd) is a antibody drug conjugate combining a HER2-targeted antibody (trastuzumab) with a topoisomerase I inhibitor by a cleavable linker. Due to incorporation of a novel linker, T-DXd achieves a higher drug-to-antibody ratio (DAR) of approximately 8 compared to first- and second-generation ADCs like Trastuzumab Emtansine (T-DM1) [18]. Importantly and in contrast to another approved ADC that targets HER2-positive breast cancer (T-DM1), the released cytotoxic drug is cell membrane permeable after it is cleaved from the linker and exhibits a bystander effect [19]. This could facilitate potent anti-cancer activity even against tumors with HER2 heterogeneity.

Trastuzumab deruxtecan has demonstrated good efficacy in second line HER2 positive advanced breast cancer (aBC; [20,21]). Also in the HER2-low cohort (immunohistochemistry (IHC) score of 1+ or an immunohistochemistry (IHC) score of 2+ and mandatory negative by in situ hybridization (ISH) (as defined in [22]) the DESTINY-Breast04 study showed improved progression-free (PFS) and overall survival (e.g., OS 23.9 months versus 17.5 months with treatment of physician’s choice [23]). Consequently, T-DXd is the standard of care in both HER2 positive and HER2 low aBC. Current phase II and III trials evaluate the efficacy of T-DXd in early breast cancer (HER2 positive: NCT05113251, NCT05704829; HER2 low: NCT04553770). However, the SURVIVE **HERoes** trial is the first study to base treatment intervention with T-DXd on an advanced liquid biopsy biomarker that is ctDNA.

## Materials and methods

### Aim, study design and sample size calculation

**Fig 1:**
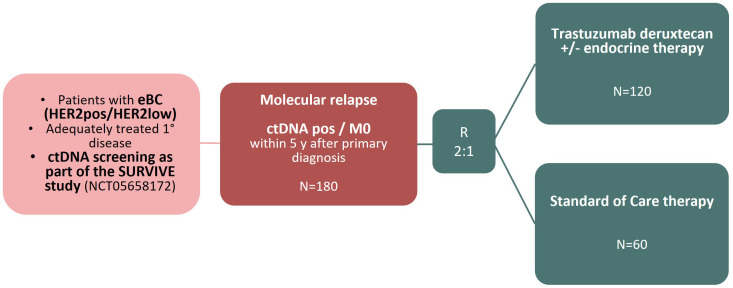
Study Schema of the SURVIVE HERoes trial. ctDNA screening is conducted as part of the SURVIVE study (NCT05658172). Upon ctDNA-positivity, staging examinations are performed, and if there are no signs of relapse (cM0), the participant becomes eligible for the SURVIVE HERoes study.

The SURVIVE **HERoes** study aims at improving the prognosis in patients with HER2 positive or HER2 low breast cancer that show a positive ctDNA test result after primary treatment. Repeated ctDNA tests are performed as part of the SURVIVE study (Standard Surveillance vs. Intensive Surveillance in Early Breast Cancer, NCT05658172 [24]). Upon positivity of ctDNA, patients receive staging examinations (CT thorax and abdomen and bone scan) [24], and if there are no signs of distant metastases (cM0), the patient becomes eligible for the SURVIVE **HERoes** trial (see Fig 1).

Based on current knowledge of the prognostic significance of ctDNA detection, including its potency to detect the efficacy of adjuvant treatment [17] the primary endpoint was determined to be ctDNA clearance rate after 12 months. Whilst IDFS or OS would be adequate endpoints with broader clinical recognition, they would need a much longer follow-up period as well as a larger sample size. Disease endpoints will, however, be included in the secondary endpoints, shown in Table 1.

**Table 1:** Study objectives and endpoints.

Primary Objective and Endpoint	ctDNA clearance rate 12 months after randomization	To compare ctDNA clearance rate twelve months after randomization between patients in the experimental arm (Arm A) versus patients in the standard of care arm (Arm B). ctDNA status (positive or negative) is measured as per RaDaR assay (see 5.8).	ctDNA clearance rate at 12 months is defined as the proportion of patients with a ctDNA negative blood test result at the time point 12 months, irrespectively of ctDNA test results obtained at other time points before: $\frac{Patients\;with\;negative\;ctDNA\;at\;12\;months}{all\;patients}$
Secondary Objectives and Endpoints	**Overall survival (OS)**	To compare OS between patients in the experimental arm (Arm A) versus patients in the standard of care arm (Arm B).	OS is defined as time from randomization until death from any cause. If a patient is not known to have died, OS is censored at the date of last contact. OS is assessed using long-term follow-up data from the SURVIVE study.
	**Invasive disease-free survival (IDFS)/ Secondary endpoint of special interest**	To compare IDFS between patients in the experimental arm (Arm A) versus patients in the standard of care arm (Arm B).	IDFS is defined as time from randomization until first IDFS event, including any invasive ipsilateral, regional, contralateral, and distant disease recurrence, second primary tumors, or death from any cause as event (non-invasive, in-situ cancer events are excluded). If a patient has not had an event, IDFS is censored at the date of last adequate tumor assessment. In addition to median IDFS, the 24 months IDFS rate will be calculated.
	**Distant disease-free survival (DDFS)**	To compare DDFS between patients in the experimental arm (Arm A) versus patients in the standard of care arm (Arm B).	DDFS is defined as time from randomization until first DDFS event including metastasis, second primary tumors and death from any cause as event. If a patient has not had an event, DDFS is censored at the date of last adequate tumor assessment
	**Distant recurrence-free survival (DRFS)**	To compare DRFS between patients in the experimental arm (Arm A) versus patients in the standard of care arm (Arm B).	DRFS is defined as time from randomization until first DRFS event including metastasis and second primary tumors; death from any cause is not included as event. If a patient has not had an event, DRFS is censored at the date of last adequate tumor assessment. If a patient has died, DRFS is censored at the date of death.
	**Breast cancer specific survival (BCSS)**	To compare BCSS between patients in the experimental arm (Arm A) versus patients in the standard of care arm (Arm B).	BCSS is defined as time from randomization until breast cancer associated death of the patient. If a patient is not known to have died, BCSS is censored at the date of last contact. If a patient has died for reasons not associated with breast cancer (by clinical assessment), BCSS is censored at the date of death.
	**Invasive breast cancer free survival (IBCFS)**	To compare IBCFS between patients in the experimental arm (Arm A) versus patients in the standard of care arm (Arm B).	IBCFS is defined as time from randomization until first IBCFS event, including any invasive ipsilateral, regional, contralateral and distant disease recurrence or death from any cause as event (non-invasive, in-situ cancer events and second primary tumors are excluded). If a patient has not had an event, IBCFS is censored at the date of last adequate tumor assessment.
	**ctDNA clearance rate after 3, 6, 9, 15, 18, 21 and 24 months**	To compare ctDNA clearance rate 3, 6, 9, 15, 18, 21 and 24 months after randomization between patients in the Experimental arm (Arm A) versus patients in the Standard of care arm (Arm B).	ctDNA clearance rate is defined as the proportion of patients with a ctDNA negative blood test result at a given time point, irrespectively of ctDNA test results obtained at other time points before.
	**Quality of life (QoL)**	To compare QoL between patients in the experimental arm (Arm A) versus patients in the standard of care arm (Arm B).	QoL will be monitored and assessed in both groups using the two questionnaires EORTC QLQ-C30 and PA-F12, which must be completed before infusion of cycle 1 and at 3, 6, 9, 12, 15, 18, and 24 months after randomization.
	**Safety and tolerability of T-DXd**	To assess safety and tolerability of treatment with T-DXd and to compare it between patients in the experimental arm (Arm A) and patients in the standard of care arm (Arm B).	Safety and tolerability of study treatments will be assessed based on the frequencies and grades of serious adverse events (SAEs) and adverse events (AEs) during the course of the study. AE of special interest will include ILD (interstitial lung disease), CHF (chronic heart failure) and left ventricular dysfunction.

The study is designed as a two-arm parallel, randomized superiority trial. Based on the primary objective of the study, the following two-sided hypotheses will be tested:

Null hypothesis H_0_:The ctDNA clearance rate 12 months after randomization is not different between the experimental arm A and the standard arm B.

Alternative hypothesis H_1_:The ctDNA clearance rate 12 months after randomization is different between the experimental arm A and the standard arm B.

To detect a clinically meaningful difference in ctDNA clearance rate 12 months after randomization of 25% (or more) between the two study arms, given a 2:1 randomization, an anticipated ctDNA clearance rate in the standard arm of 35% (based on ctDNA clearance rates obtained in studies in at least similar – although not equal – clinical settings ranging from approximately 20–50% [25,26]), and an anticipated dropout rate of 20%, approximately 180 patients (120 patients in the experimental arm A and 60 patients in the standard arm B) will be required to achieve at least 80% power at a 2-sided significance level of α = 0.05 using a chi-square test to compare two independent proportions.

### Study Population

Participants eligible for enrolment into the SURVIVE **HERoes** study are participants of the SURVIVE study. As such, they have completed primary therapy according to clinical routine, including a R0 surgeryand possible (neo-)adjuvant therapy and radiotherapy. To be eligible for inclusion into the SURVIVE main study (NCT05658172), participants must have medium to high risk of recurrence, defined as a large tumor (≥T3), positive lymph nodes (> N1mi), high genomic risk or indication to chemotherapy. Simultaneously, the other inclusion/ exclusion criteria of the SURVIVE study must be fulfilled.

Patients can be enrolled into SURVIVE immediately following the completion of primary therapy, and up to 3 years thereafter (up to 5 years for HR + , HER2- tumors). In the SURVIVE main study, 50% of patients receive intensive surveillance including 3-monthly ctDNA tests during year 1–3 and 6-monthly ctDNA tests during year 4 and 5 (for more details see [24]). In case of positivity of ctDNA, SURVIVE participants receive staging examinations. In case of no signs of local or distant recurrence, participants become eligible for enrolment into SURVIVE **HERoes**.

As for SURVIVE **HERoes**, participants must be either HER2-positive or HER2-low. Hormone receptor positive and negative patients are allowed. Inclusion during all available adjuvant treatment options is permitted (except for (neo-)adjuvant primary chemotherapy).

Participants for the SURVIVE **HERoes** trial must show positivity for ctDNA no longer than 8 weeks (56 days) before randomization, whilst staging examinations (minimum CT thorax and abdomen, and additional bone scan, performed within 6 weeks (42 days) before randomization) must not show evidence of relapse (cM0). Participants must be eligible for treatment with trastuzumab deruxtecan (see Table 2).

**Table 2:** Main inclusion and exclusion criteria.

Main Inclusion Criteria
Written informed consent for all study procedures according to local regulatory requirements prior to beginning specific protocol procedures.
Females or males, ≥ 18 years and ≤ 75 years of age.
Invasive breast carcinoma as revealed by local pathology that is either: HER2-positive defined as an immunohistochemistry (IHC) score of 3+ and/or positive by in situ hybridization (ISH) in Her2 2 + tumors (as defined in 2018 American Society of Clinical Oncology – College of American Pathologists [ASCO-CAP] guidelines) HER2-low defined as an immunohistochemistry (IHC) score of 1+ or an IHC score of 2+ with a mandatory negative in situ hybridization (ISH), as defined in 2018 American Society of Clinical Oncology – College of American Pathologists [ASCO-CAP] guidelines.
Complete resection of the tumor with resection margins free of invasive carcinoma (R0).
Participation in the SURVIVE study and evidence of molecular relapse (as assessed based on a positive ctDNA result obtained in the SURVIVE-study)
No evidence of metastatic relapse as revealed by a CT-scan (Abdomen/Chest) and a bone scan that must be performed within 8 weeks before randomization (M0).
Completion of surgery, (neo-)adjuvant chemotherapy (if applicable) and radiation therapy (if applicable, whichever occurred last) at least 6 months before randomization.
Adjuvant/Postneoadjuvant treatment with Trastuzumab, Pertuzumab, T-DM1, Capecitabine, Pembrolizumab, and Olaparib must be discontinued upon randomization into Arm A (treatment with trastuzumab deruxtecan). The washout periods must be complied with. Endocrine therapy (i.e., Tamoxifen, Letrozol, Anastrozol, Fulvestrant or Exemestane) can be administered simultaneously to treatment with trastuzumab deruxtecan.
Known HR status, per local laboratory assessment, as defined by ASCO-CAP guidelines (≥1%): HR-positive status defined by either positive estrogen receptor (ER) and/or positive progesterone receptor (PR) status. HR-negative status defined by both known negative ER and known negative PR
Left ventricular ejection fraction (LVEF) ≥ 50% within 28 days prior to randomization
Eastern Cooperative Oncology Group (ECOG) performance status of 0 or 1 at Screening
Adequate organ and bone marrow function
Adequate treatment wash-out periods for specific anti-cancer or anti-infectious treatments
Safe contraceptive methods for both men and pre- and perimenopausal female patients

### Study interventions and randomization

180 patients will be enrolled in the SURVIVE **HERoes** study. Randomization will be into one of the two treatment arms in a 2:1 ratio (trastuzumab deruxtecan: Standard of Care (SoC) therapy). The randomization will be stratified by the following 2 stratification factors:

- HER2 status (positive vs. low), for definition see Main Inclusion Criteria.
- Hormonal status (positive versus negative), HR positive defined as estrogen and/or progesterone receptor ≥1%

After a screening period of 28 days maximum, treatment of either trastuzumab deruxtecan or SOC therapy will commence. In case of randomization to SOC therapy, the pre-existing therapy must not be changed. In case of no oncological treatment at the time of screening, SOC therapy will comprise active surveillance only.

**Fig 2:**
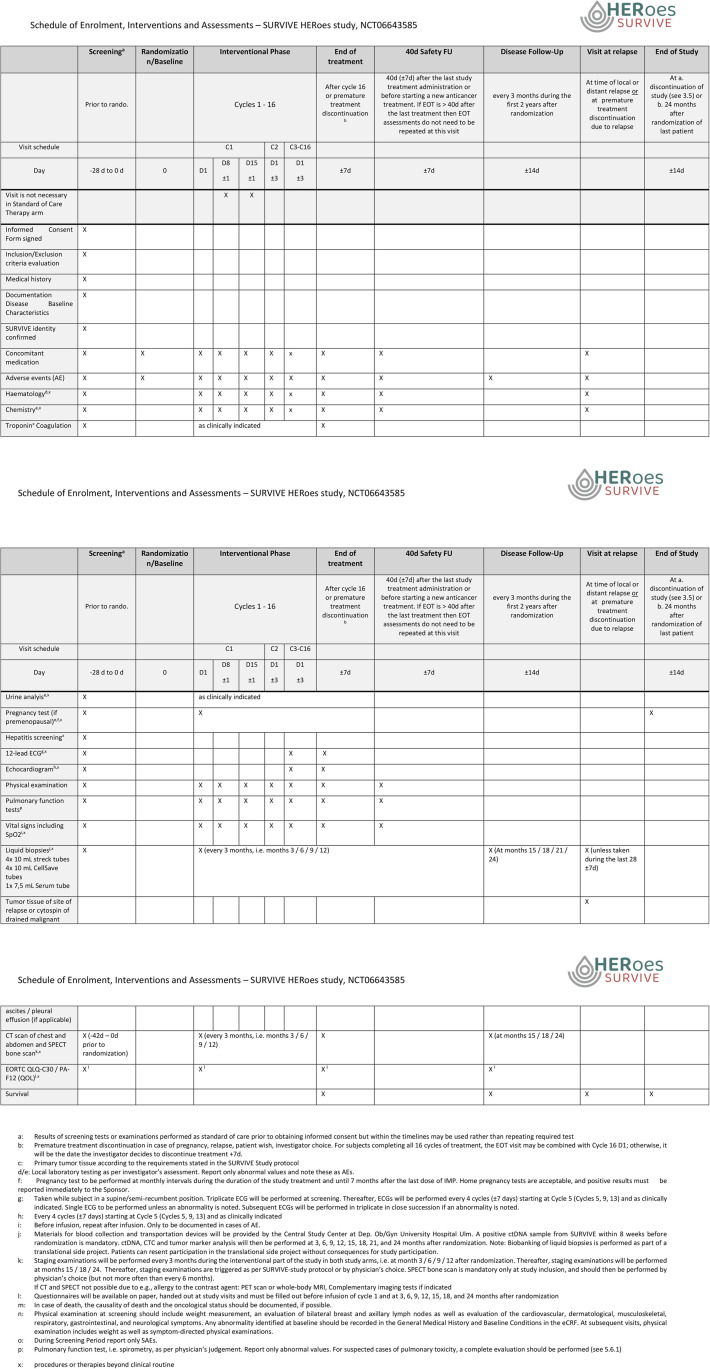
Schedule of Enrolment, Interventions and Assessments.

In the T-DXd arm, treatment will comprise 16 cycles of trastuzumab deruxtecan 5.4 mg/kg d1, repeated every 21 days. In the case of hormone receptor positivity (≥1%), endocrine therapy is administered in parallel, according to local guidelines. Study examinations aim at securing safe administration (see Fig 2).

In case of toxicities, dose modifications, delays and adjourns of therapy can be required. For specifics, please see the study protocol. The dose reduction levels are as following: 5.4 mg/kg body weight → 4.4 mg/kg body weight → 3.2 mg/kg body weight.

Due to the recent data on potentially fatal ILD (interstitial lung disease) [27,28], special attention was given to management of pulmonary and cardiac toxicities (shown in supplementary materials (S1–S2 Table). Patients are thoroughly educated on pulmonary and cardiac side effects, are presented with a lay language pocket card and are required to sign a specific informed consent form depicting the symptoms of pulmonary and cardiac toxicities. Study centers receive thorough and repeated teaching on side effect management. All events of ILD/pneumonitis, regardless of severity or seriousness, are followed until resolution.

**Fig 3:**
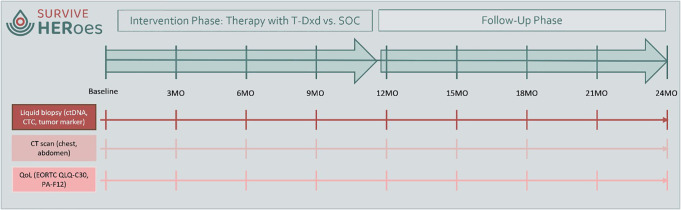
Interventions of the SURVIVE HERoes study.

Study interventions to assess efficacy are performed in parallel in both study arms and include ctDNA analyses at baseline (ctDNA positivity being an inclusion criterion), in month 3, 6, 9, 12, 15, 18, 21 and 24 as well as at time of relapse or end of study (see Fig 3). A tumor-informed approach will be utilized (RaDaR test, NeoGenomics, Florida, USA) – for specifics please refer to the SURVIVE study protocol (NCT05658172).

Simultaneously, circulating tumor cells (CTC) are assessed at the depicted ctDNA timepoints. As a translational side project, patients are asked for consent for analyses of tumor material in the case of relapse. Translational research will aim at identifying modes of defense mechanisms and carcinogenic evolution.

CT scans of chest and abdomen are performed at baseline (with no signs of relapse being an inclusion criterion), as well as parallel to the ctDNA tests in months 3, 6, 9, 12, 15, 18, and 24. There is no need for RECIST assessment (see Fig 3). A bone scan is performed at baseline.

### Statistical Analysis and data management plan

The results regarding the **primary endpoint** ctDNA clearance rate 12 months after randomization (defined as the proportion of patients with a ctDNA negative blood test result as determined 12 months after randomization) will be described using frequency tables presenting absolute and relative frequencies of ctDNA clearance together with the appropriate confidence intervals. The comparison of ctDNA clearance rate between participants in the Experimental arm (Arm A) and participants in the Standard of care arm (Arm B) will be performed using non-parametric statistical methods appropriate for the analysis of frequencies and rates (chi square test for two independent proportions). For all participants who are dropouts (lost to follow-up, withdrawal of consent), have a disease recurrence as confirmed by imaging or die within the first 12 months, or have no ctDNA assessment 12 months after randomization for other reasons, the primary outcome parameter ctDNA clearance after 12 months will be regarded as missing value and treated as such in the primary analysis.

An adjusted multivariable binary logistic regression analysis with ctDNA clearance after 12 months (yes/no) as binary response variable and randomization arm (experimental arm/standard arm), the two stratification factors HER2 status (positive/low) and hormonal status (positive/negative), as well as additional prognostic factors (to be determined in the SAP) as predictor variables will be performed as secondary analysis for the primary objective.

To test for the possibility that taking a ctDNA sample can be informed by group and outcome and thus for a potential bias imposed by intercurrent events leading to missing values, pre-planned sensitivity analyses regarding the primary endpoint will be conducted. One sensitivity analysis will be performed with early recurrences (before the 12-month ctDNA assessment) being treated as ctDNA positive rather than being regarded as missing values. More details of the sensitivity analyses will be set forth in the SAP (statistical analysis plan).

All analyses regarding the primary endpoint will be based on the ITT (intention to treat) set.

A preplanned **interim analysis** for superiority will be conducted after 90 patients have reached the timepoint of the primary endpoint (i.e. ctDNA clearance 12 months after randomization). The Lan-DeMets alpha-spending function with O’Brian-Fleming type stopping boundary is used to control for the overall two-sided type I error probability set at 0.05. The two-sided p-value obtained by the interim analysis has to be less than 0.013 (the efficacy stopping boundary) to reject the null hypothesis of no difference in ctDNA clearance rate after 12 months between the two randomization arms and to conclude superiority (i.e., a higher ctDNA clearance rate) in the experimental arm. If the p-value obtained in the interim analysis does not cross the efficacy stopping boundary, the p-value obtained in the final analysis has to be less than 0.044 to reject the null hypothesis of no difference in ctDNA clearance rate after 12 months.

All analyses regarding the **secondary objectives** will have exploratory character only.

### Regulatory, dissemination and timeline

**Fig 4:**
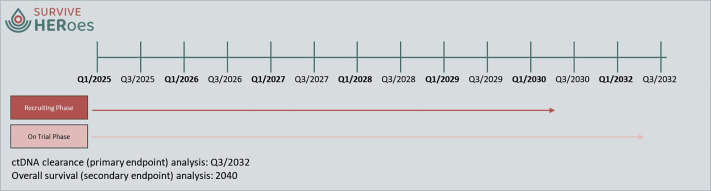
Timeline and anticipated dates of endpoints.

The trial has been registered via the CTIS system of the European Medicines Agency (EMA) (EU CT Number: 2024-516173-76-01). Approval by the Ethics committee and competent authority (EC/CA) has been obtained in December/ January 2024/ 2025. The recruitment began in 04/2025 and is expected to continue for 5 years. The last visit of the last patient will thus be in Q2/2032 (see Fig 4).

The study is registered at clinicaltrials.gov (**NCT06643585**).

There are 100 planned SURVIVE study centers, of which 50 will be selected as SURVIVE **HERoes** study centers. The selection will be based on experience with clinical trials and geographic distribution across Germany to ensure that every patient lives within a reasonable distance of a SURVIVE **HERoes** study center.

## Discussion

### Secondary adjuvant treatment option

The emergence of ctDNA as a biomarker offers the potential to tailor adjuvant treatment more precisely to individual risk profiles. For instance, the prognostic implications of pathological complete remission (pCR) have already led to the adaptation of adjuvant therapy based on the response to neoadjuvant chemotherapy [29]. This paradigm has enabled both escalation and de-escalation of adjuvant therapies [30]. Similarly, ctDNA, with its potential as a robust prognostic biomarker, could guide secondary adjuvant therapy, in a similar way to non-pCR in post-neoadjuvant treatment strategies.

While SURVIVE **HERoes** emphasizes therapy escalation, ctDNA could also facilitate de-escalation in patients undergoing toxic treatment regimens who lack ctDNA positivity, potentially sparing them unnecessary side effects. In the MonarchE study, only 45% of patients receiving endocrine therapy alone, and 32.5% of those receiving endocrine therapy combined with Abemaciclib, reported not being bothered by treatment side effects after three months [31]. Incorporating repeated ctDNA assessments and monitoring of ctDNA clearance during treatment into adjuvant therapy decision-making could help tailor therapies more precisely, potentially reducing the burden of side effects for patients [17]. This underscores the transformative role ctDNA could play in refining and personalizing follow-up care and secondary adjuvant treatment in breast cancer management.

### T-DXd as secondary adjuvant therapy

Trastuzumab deruxtecan has proven outstanding efficacy in both HER2 positive and HER2 low breast cancers in the metastatic setting [21,23] and is tested in the adjuvant and neoadjuvant setting (DESTINY-Breast05, NCT04622319; DESTINY-Breast11, NCT05113251; ADAPTHER-IV, NCT05704829; NCT04553770; SHAMROCK, NCT05710666; EXTEND, NCT06548178). However, side effects must be discussed in equal depth. Whilst hematological and gastrointestinal symptoms associated with T-DXd treatment present a similar, if not more favorable, side effect profile compared to physician’s choice chemotherapy [21,23], careful attention must be given to pulmonary side effects, which can be fatal. Interstitial lung disease (ILD) in patients treated with T-DXd has an incidence of 11.4–15.4% (grade 5, i.e., fatal, 0–2.2% of all treated patients) [21,23,32,33] and a median time of 82 days (IQR (interquartile range) 49–192 days) was observed from therapy initiation to diagnosis of ILD [27]. Incidence was higher in patients with preexisting lung conditions, baseline SpO2 below 95%, smoking, prior chest irradiation and renal impairment [28,32,33].

This study was designed with careful consideration of these potentially fatal side effects. The following steps are undertaken:

1. Exclusion of participants with anticipated high rate of ILD, e.g., in case of preexisting lung conditions
2. Thorough teaching of study personnel during study initiation and beyond
3. Extensive patient education (informed consent form with separate sheet for ILD, lay language pocket card, etc.)

Nevertheless, the question arises whether treating participants without any radiographic signs of tumor with a potentially toxic drug is ethically justified. As tumor-informed ctDNA tests have recently been shown to exhibit high specificity [15], a positive test result indicates an already limited prognosis for the patient, with a mean lead time from positive test to overt cancer recurrence of 10.8 months [15]. During this time, molecular relapse presents a unique therapeutic window. Clinical benefit rate in the DESTINY-Breast03 (HER2 positive aBC) and DESTINY-Breast04 (HER2 low aBC) was 76.1 and 70.2%, respectively [21,23]. In the DESTINY-Breast06 trial (HR positive aBC without prior chemotherapy) an even higher clinical benefit rate was observed (76.6%) [34]. If these figures are projected onto the MRD setting, the potential of saved patient years is substantial. Given the substantial potential benefits of secondary adjuvant treatment with T-DXd in terms of considerably increased disease-free survival (perhaps even cure) in the setting of molecular relapse without overt metastases, the benefit/risk ratio is deemed highly favourable. The ethics committee overseeing this trial endorsed our approach by approving the proposed study.

### Limitations of the study design

This study uses ctDNA clearance rate after 12 months as an innovative primary endpoint. In the small field of therapy escalation studies in the MRD setting, 5/9 studies have decided on a ctDNA based endpoint (LEADER NCT03285412; MIRaDoR NCT05708235; ASPIRA NCT04434040; KAN-HER2 NCT05388149 and the SURVIVE **HERoes** study), while 4/9 opted for a disease endpoint (disease-free survival (DFS), distant metastasis-free survival (DMFS) or relapse-free survival (RFS); Treat ctDNA NCT05512364; TRAK-ER NCT04985266, DARE NCT04567420 and PERSEVERE NCT04849364). While disease endpoints may represent a more clinically meaningful outcome, ctDNA metrics—particularly clearance—could serve as a valuable surrogate parameter, while reducing follow-up time and thus study costs. In the SURVIVE **HERoes** trial, a variety of therapies are allowed as Standard of Care treatment in Arm B. This may also include no treatment at all if the patient did not receive any treatment at the time of ctDNA positivity. The considerable heterogeneity of the SoC cohort was accepted based on the assumption that the current treatment regimen has led to the emergence of ctDNA. In continuation, we hypothesize that an active (residual) tumor is to be suspected and a change of treatment regimen is a favorable recommendation for all ctDNA positive patients. Heterogeneity may present challenges in interpreting the results of the study, in particular when the primary endpoint is not reached. Subpopulations of patients (i.e., HER2 positive patients, patients with an interval between adjuvant therapy and ctDNA positivity, etc.) who might benefit most from secondary adjuvant treatment with trastuzumab deruxtecan may be difficult to identify. Albeit the aforementioned limitations, the present study proposes a pioneering therapeutic strategy of secondary adjuvant treatment in a new therapeutic window based on a novel biomarker. Consequently, the observed limitations were deemed acceptable in order to test the hypothesis: Will targeting MRD be the last chance for cure?

## Supporting information

S1 Table Toxicity management guidelines for AE of special interest (interstitial lung disease (ILD) and cardiac toxicity, i.e., chronic heart failure (CHF) and left ventricular dysfunction). (DOCX)

S2 Table Spirit Checklist. (DOCX)

S1 File 241121 SURVIVE HERoes Protokoll V01.4 ohneLogos. (DOCX)
