# Supplementary material for: Study Protocol of SURVIVE HERoes (NCT06643585): Trastuzumab Deruxtecan for molecular relapse in HER2+/ Low early breast cancer with ctDNA positivity after primary therapy
Source: PLoS One. 2025 Nov 19;20(11):e0322156. doi: 10.1371/journal.pone.0322156 (PMC12629461; doi:10.1371/journal.pone.0322156)
Supplement: S1 Table — (DOCX) [file pone.0322156.s001.docx]

**S1 - Supporting Information Table 1: Toxicity management guidelines for AE of special interest (interstitial lung disease (ILD) and cardiac toxicity, i.e. chronic heart failure (CHF) and left ventricular dysfunction).**

| **Cardiac Toxicity** | |
| --- | --- |
| Symptomatic congestive heart failure (CHF) | Discontinue subject from study treatment |
| Decrease in Left ventricle ejection fraction (LVEF) 10-20% (absolute value), but LVEF > 45% | Continue treatment with trastuzumab deruxtecan |
| LVEF 40% to ≤ 45% and decrease is < 10% (absolute value) from baseline | Continue treatment with trastuzumab deruxtecan  Repeat LVEF assessment within 3 weeks |
| LVEF 40% to ≤ 45% and decrease is 10-20% (absolute value) from baseline | Interrupt trastuzumab deruxtecan dosing  Repeat LVEF assessment within 3 weeks.  If LVEF has not recovered to within 10% (absolute value) from baseline, discontinue subject from study treatment  If LVEF recovers to within 10% from baseline, resume treatment with study drug |
| LVEF < 40% or > 20% (absolute value) drop from baseline | Interrupt trastuzumab deruxtecan dosing  Repeat LVEF assessment within 3 weeks.  If LVEF < 40% or > 20% drop from baseline is confirmed, discontinue subject from study treatment  If LVEF has recovered to > 40% and decrease is < 20% from baseline follow appropriate guidance above. |

| **Pulmonary Toxicity** | | Work-up of suspected ILD/pneumonitis:  If a subject develops radiographic changes potentially consistent with ILD/pneumonitis or develops an acute onset of new or worsening pulmonary or other related signs/symptoms such as dyspnea, cough or fever, rule out ILD/pneumonitis   - Evaluations should include: - CT of chest - Pulmonologist consultation (Infectious Disease consultation as clinically indicated) - Blood culture and CBC. Other blood tests could be considered as needed - Consider bronchoscopy and bronchoalveolar lavage if clinically indicated and feasible - Pulmonary function tests (including forced vital capacity (FVC) and carbon monoxide (CO) diffusing capacity and pulse oximetry - Arterial blood gases if clinically indicated - One blood sample collection for PK analysis as soon as ILD/pneumonitis is suspected, if feasible - Covid-19 test, if feasible   Other tests could be considered, as needed.  If the AE is confirmed to have an etiology other than treatment-related ILD/pneumonitis, follow the management guidance outlined in the “Other Non-Laboratory Adverse Events” dose modifications.  If another etiology for the AE cannot be identified and it could be related to trastuzumab deruxtecan, then follow the ILD/pneumonitis management guidance as outlined below.  All events of ILD/pneumonitis, regardless of severity or seriousness, should be followed until resolution | |
| --- | --- | --- | --- |
| Grade 1 | | Management:   - Monitor and closely follow-up in 2 to 7 days for onset of clinical symptoms and pulse oximetry then weekly as indicated - Consider follow-up imaging in 1-2 weeks (or as clinically indicated). - Consider starting systemic steroids (for example at least 0.5 mg/kg/day prednisone or equivalent) until improvement, followed by gradual taper over at least 4 weeks. - If worsening of diagnostic observations despite initiation of corticosteroids, then follow Grade 2 guidelines.*   Dose modification:  The administration of trastuzumab deruxtecan must be interrupted. Trastuzumab deruxtecan can be restarted only if the event is fully resolved to Grade 0:   1. If resolved in ≤ 28 days from day of onset, maintain dose 2. If resolved in > 28 days from day of onset, reduce dose 1 level   However, if the event grade 1 ILD/pneumonitis occurs beyond cycle day 22 and has not resolved within 49 days from the last infusion, the drug should be discontinued.  *If a subject is asymptomatic, then the subject should still be considered as Grade 1 even if steroid treatment is given | |
| Grade 2 | | Dose Modification:  Permanently discontinue subject from study treatment.  Management:   - Promptly start and treat with systemic steroids (for example, at least 1mg/kg/day prednisone or equivalent) - gradual taper over at least 4 weeks. - Monitor symptoms closely. - Re-image as clinically indicated. - If worsening or no improvement in clinical or diagnostic observations in 3-5 days, Consider increasing dose of steroids (for example, 2 mg/kg/day prednisone or equivalent) and administration may be switched to intravenous (e.g. methylprednisolone). - Re-consider additional work-up for alternative etiologies as described above.  1. Escalate care as clinically indicated. | |
| Grade 3 or 4 | Dose modification:  Permanently discontinue subject from study treatment.  Management:   - Hospitalization required. - Promptly initiate empiric high-dose methylprednisolone IV treatment (for example, 500-1000 mg/day for 3 days), followed by at least 1.0 mg/kg/day of prednisone (or equivalent) for at least 14 days followed by a gradual taper over at least 4 weeks. - Re-image as clinically indicated.   - If still no improvement within 3 to 5 days, Re-consider additional work-up for alternative etiologies as described above.   - Consider other immuno-suppressants and/or treat per local practice. | |  |
